# Utilizing the Social Determinants of Health Model to Explore Factors Affecting Nurses’ Job Satisfaction in Saudi Arabian Hospitals: A Systematic Review

**DOI:** 10.3390/healthcare11172394

**Published:** 2023-08-25

**Authors:** Ali Hudays, Fay Gary, Joachim G. Voss, Amy Y. Zhang, Alya Alghamdi

**Affiliations:** 1 Frances Payne Bolton School of Nursing, Case Western Reserve University, Cleveland, OH 44106, USA; fxg21@case.edu (F.G.); jgv20@case.edu (J.G.V.); amy.zhang@case.edu (A.Y.Z.); 2 Community, Psychiatric, and Mental Health Nursing Department, College of Nursing, King Saud University, Riyadh 11437, Saudi Arabia; aalgamdii@ksu.edu.sa

**Keywords:** job satisfaction, nurses, Saudi Arabia, social determinants of health, workplace

## Abstract

The purpose of this systematic review was to explore factors affecting nurses’ job satisfaction in Saudi Arabian hospitals by utilizing the social determinants of a health model. We conducted a systematic review using three databases (PubMed, PsychINFO, and CINAHL) following the Preferred Reporting Items for Systematic Reviews and Meta-Analysis. A total of 235 studies were screened. Of these studies, nine met the inclusion criteria. The studies were appraised using the Joanna Briggs Institute checklist tool. The majority of studies reported that salary, years of experience, nationality, and marital status were factors affecting nurses’ job satisfaction. Gender and educational level did not impact job satisfaction for male and female nurses. Overall, the review highlighted some knowledge gaps in the assessment of the impact of social determinants of health regarding gender and educational level on nurses’ job satisfaction. Further research is needed to address this knowledge gap.

## 1. Introduction

Job satisfaction can be defined as the extent to which people feel either positively or negatively about their work [1]. Job satisfaction can also be defined as the degree to which an employee is satisfied with the benefits they receive from their work, especially when it comes to intrinsic motivations [2]. It is a complicated concept that has been studied in many different fields, such as nursing, business, psychological science, and sociology [3]. The concept of job satisfaction and its dimensions have been largely shaped by a number of classical works throughout history; these works include Herzberg’s Two-Factor Theory, Hackman and Oldham’s Job Characteristics Model, McCloskey and McCain’s Nurses’ Job Satisfaction Scale, and Aiken’ Magnet Hospital study [4,5,6,7].

Herzberg’s Theory of Job Satisfaction and Dissatisfaction was developed in the 1950s by Frederick Herzberg [4]. It posits that job satisfaction is affected by two components: motivators (such as recognition, responsibility, and advancement), and hygiene factors (such as salary, working environment, and company policies) [4]. Numerous studies have been conducted to explore the role of these two components in job satisfaction in the nursing profession and to identify the specific motivators and hygienic factors that influence job satisfaction [8,9,10,11].

Hackman and Oldham developed the Job Characteristics Model, which suggests that certain job characteristics contribute to job satisfaction [5]. These characteristics include skill variety, task identity, task significance, autonomy, and feedback. Several studies have applied this model to nursing and highlighted the importance of these job characteristics in determining nurses’ job satisfaction, such as [12,13,14,15].

McCloskey and McCain developed a widely used instrument called the Nurses’ Job Satisfaction Scale (NJSS) to measure job satisfaction specifically among nurses [7]. This scale assesses various dimensions of job satisfaction, including autonomy, professional status, interaction, and task requirements [7]. The NJSS has been employed in numerous studies to measure and understand job satisfaction among nurses; for example, [16,17,18,19].

The Magnet Hospital study conducted by Aiken and colleagues explored the relationship between nursing job satisfaction and organizational characteristics [6]. The study identified several factors associated with higher job satisfaction among nurses, including supportive nurse–physician relationships, participative management style, and opportunities for professional development [6]. These classical works provide valuable insights into the dimensions and determinants of job satisfaction among nurses. However, it is important to note that the field of nursing and healthcare is constantly evolving, and new research continues to contribute to our understanding of job satisfaction and its dimensions among nurses.

In Saudi Arabia, nurses provide up to 80% of patient care, which means that nurses’ competencies substantially affect patient outcomes [20]. The Saudi healthcare system is significantly challenged due to the considerable shortage of healthcare providers, particularly nurses, despite the 2030 Vision launched by the Saudi government for implementing Saudization [21].

The 2018 annual report of the Ministry of Health showed that nurses from outside of Saudi (expat) nurses comprise about 64% of the Saudi healthcare workforce, compared to Saudi nurses, who account for 36% of the workforce [22]. It is estimated that the Saudi healthcare system will need about 100,000 additional nurses by 2030 [21]. Throughout the country, the Ministry of Health operates 2361 primary healthcare centers and 282 hospitals, which are responsible for providing primary, secondary, and tertiary care [23]. Therefore, the aforementioned shortage of nurses has led to the frustration and dissatisfaction of nurses with their workplace as a result of the heavy workloads assigned to them [24]. This dissatisfaction is shared with nurses from other countries. A study conducted in five countries evaluating nurses’ job satisfaction showed that the rate of job dissatisfaction in the United States of America was the highest (41%) compared to those in Scotland, the United Kingdom, Canada, and Germany, at 38%, 36%, 33%, and 17%, respectively [25,26]. Furthermore, the primary predictor of job dissatisfaction among nurses in these countries was work overload [25].

Nurses’ dissatisfaction has consequences for the healthcare system, including increased rates of turnover among nurses, which negatively impact the healthcare quality provided to patients. An integrative review conducted by [27] that included studies between 1990 and 2017 revealed an increase in nurse turnover in Saudi Arabia, from 17% in 2008 to 60% in 2014, in both the public and private health sectors. The review also identified nurses’ demographics, satisfaction, and job-related factors as determinants of high nurse turnover. These factors include gender, marital status, living with a spouse, educational attainment, monthly salary, years of experience, and nationality. However, we know little about the social context of these nurses. Thus, the social determinants of health (SDOH) framework chosen to understand which factors influenced nurses the most to feel dissatisfied.

**Figure 1:**
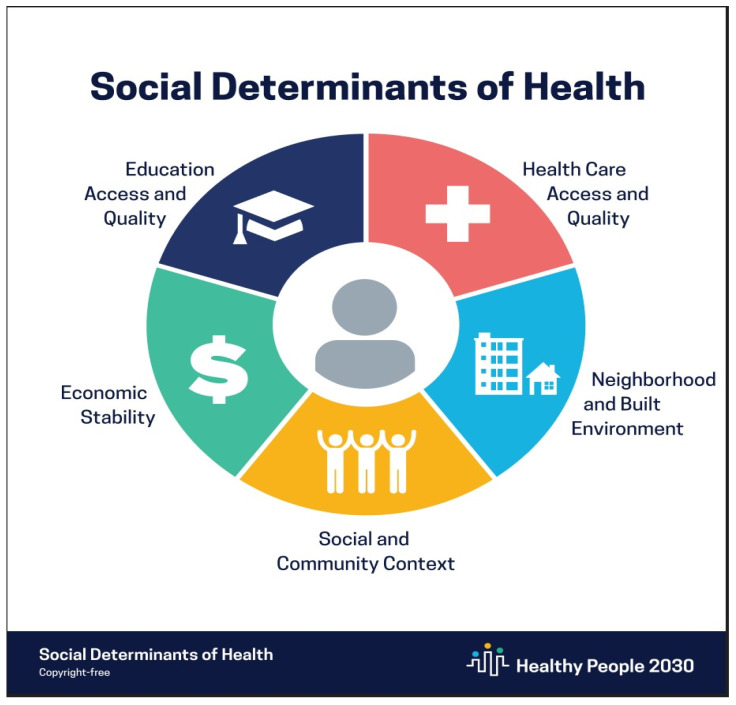
The social determinants of health.

According to the World Health Organization, SDOH is defined as “A subset of the inherited conditions in which people are expected to live, grow, work, and age” [28]. The SDOH is categorized into five key domains based on the Healthy People 2020 campaign, led by the Office of Disease Prevention and Health Promotion: economic stability, education, healthcare access, neighborhood and built environment, and social and community context [29] (Figure 1). Providing high-quality care requires health system policymakers to understand nurses’ circumstances and to balance clinical knowledge with social needs. These social factors, when neglected, may limit the number of clinical duties assigned to nurses. Therefore, nurses must be adequately educated about SDOHs and have sufficient knowledge and tools to minimize the impact of SDOHs on health outcomes.

Several studies have reported factors that act as barriers to nurses’ satisfaction. The results of a literature review of 20 studies published between 2007 and 2012 showed that age, gender, education, and organizational and work environment factors can influence job satisfaction levels [30]. Moreover, a comprehensive literature review was conducted to review the barriers and motivators of job satisfaction among nurses in Saudi Arabia from 2006 to 2016 [31]. Most of the results indicated that the factors that act as barriers to the satisfaction of nurses include salary, lack of support for stress relief, head nurses’ leadership and professional support, prospects for promotion, and a lack of educational opportunities [31].

Another study conducted by Al-Dossary et al. [32] measured the job satisfaction of 189 nurses working in a teaching hospital in Saudi Arabia, and found that pay, fringe benefits, contingent rewards, and operating conditions were sources of dissatisfaction among the nurses, while age, gender, and level of education did not have an influence on the nurses’ job satisfaction. Moreover, a study conducted by Hamaideh [33] on 181 mental health nurses in Jordan explored the relationships between burnout, social support, and job satisfaction. The results showed high levels of emotional exhaustion and moderate levels of depersonalization and personal accomplishment among mental health nurses were related to dissatisfaction [33]. The study further indicated that low levels of education were a significant factor that increased burnout among nurses and recommended that nurses should receive more advanced education [33]. When nurses received educational opportunities from their organizations, it positively reflected on the organizations. By contrast, not having opportunities or having fewer opportunities mostly lowered nurses’ motivation within their organizations.

The factors identified in the above findings could be attributed to the SDOH framework. However, there is a gap in the literature, as no studies have used the SDOH framework to address the determinants of job satisfaction among nurses in Saudi Arabia. An exception is one study conducted by Almujadidi et al. [34] on 17 primary healthcare physicians working at King Khalid University Hospital in Saudi Arabia to identify barriers and enablers for addressing SDH in clinical settings in Saudi Arabia. The authors identified common social problems, such as financial burdens, mental health issues, and aging population difficulties, as well as a lack of action by primary care physicians to address these SDH [34]. This lack of action may be attributed to the physician’s lack of knowledge and training, the organization being under time constraints, patients being referred/followed up, patient cultural norms not understood, and physicians’ unawareness of their role in caring for patients [34]. Therefore, this review aimed to explore the barriers to nurses’ job satisfaction in Saudi Arabian hospitals by utilizing the social determinants of the health (SDOH) model and to draw conclusions that will aid in filling this research gap and enhance nurses’ knowledge about the SDOH.

### Research Question

“To what extent do social determinants impact nurses’ job satisfaction in Saudi Arabian hospitals?”

## 2. Materials and Methods

### 2.1. Search Strategy

A search strategy was conducted using the following databases: PubMed, PsychINFO, CINAHL, and Google Scholar. Table 1 provides the keywords used for the search. The keywords included “Saudi nurses”, “foreign nurses”, “staff nurses”, “social determinants”, “social barriers”, “social determinants of health”, and “job satisfaction”, “work satisfaction”. The search terms or keywords were combined using the Boolean operators “AND” and “OR”. Using different search terms and incorporating the two operators led to a focused search, which then generated relevant journals in line with the research question. Furthermore, filters were applied as search limits in the search process to generate the appropriate studies. As a method of finding relevant, scholarly, and peer-reviewed studies, the filters included studies published between January 2014 and March 2023 to obtain up-to-date evidence. Journal articles were selected by establishing eligibility criteria (Table 1).

### 2.2. Inclusion and Exclusion Criteria

In this systematic review, the inclusion criteria for selection consisted of quantitative studies in nature. All studies focused on Saudi and non-Saudi nurses. Only studies conducted in Saudi hospitals were included. Although the Preferred Reporting Items for Systematic Reviews and Meta-Analysis (PRISMA) guidelines indicate that searches should not apply limits to date or language, in this study, language was limited to English. Studies conducted outside Saudi Arabia were excluded if they focused on other health professions. Systematic reviews, books, and gray literature were excluded (Table 1).

### 2.3. Search Outcomes

**Figure 2:**
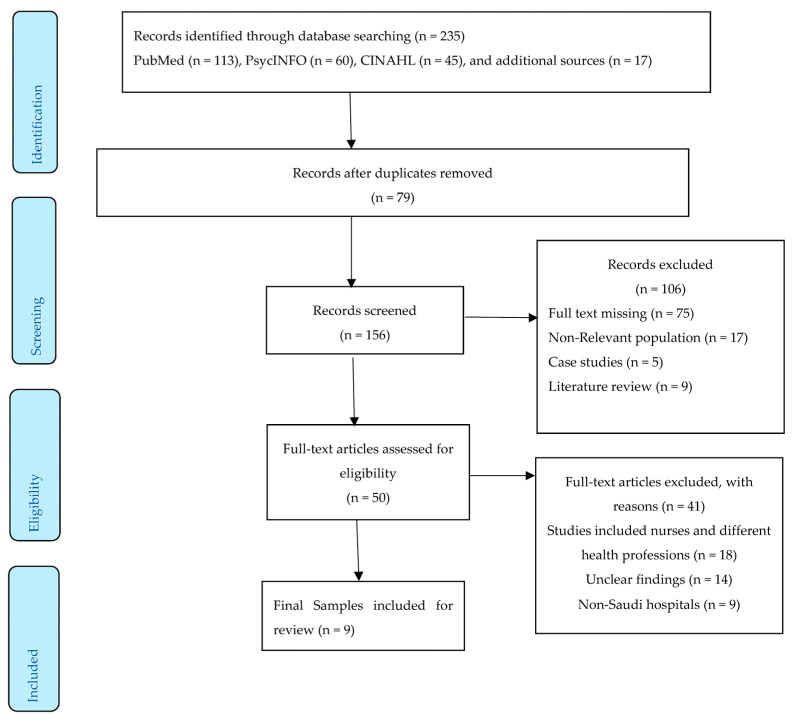
Prisma flow diagram.

Two independent reviewers (J.G.V. and A.Z.) reviewed the titles and abstracts to see if there were any relevant citations that fit the selection criteria. Two other independent reviewers (F.G. and A.H.) reviewed the e-search and provided us with a full report of all the citations that fit the pre-defined selection criteria. The data abstraction was carried out by two evaluators and the verifications were carried out by two evaluators. Any inconsistencies in the screening of the titles/abstracts and full-text articles were addressed through discussion, with a third reviewer’s adjudication if required. We used three databases (PubMed, PsychINFO, and CINAHL) and found 235 articles, which were then subjected to the selection process following the PRISMA flow diagram [35]. Figure 1 describes the entire screening process. All articles were uploaded to EndNote X9 and screened for duplicates. The initial screening of titles and abstracts resulted in the removal of non-relevant articles. A total of 50 full articles remained; however, 41 articles did not meet the inclusion criteria, resulting in a final sample of nine articles included in this review, as shown in the PRISMA diagram in Figure 2 and Table 2.

### 2.4. Data Abstraction

After including the nine studies that fulfilled the inclusion criteria for the review, data extraction was carried out utilizing a structured table that included the following categories: author’s name, country and time frame, design, tool used, setting, sample size, characteristics of participants, ages, determinants, key findings, and SDOH themes (Table 2).

**Table 2:** Characteristics of reviewed articles.

Authors, Year	Country/Time Frame	Study Design	Sample Size/Population Characteristics	Determinants	Key Findings	SDOH Themes
[36]	Saudi Arabia March 2015 to March 2016. (Riyadh)	A cross-sectional survey. Tool Used: A self-administered questionnaire	The study was conducted on (n = 364 nurses) working at two different tertiary hospitals in Riyadh city namely King Fahad Medical City (KFMC) and King Faisal Specialized Hospitals (KFSH). Male: 35 (9.6%) Female: 329 (90.4%) Ages: between 22 and 51. Nationality: Saudi (n = 9), and non-Saudi (n = 355)	-Years of experience. -Monthly income. -Gender	There was a statistically significant difference between satisfactory nurse status and years of nursing experience with (*p* = 0.025). Based on logistic regression analysis there was statistically significant difference between nurses’ satisfaction and those receive less than 5000 and 5000–10,000 Saudi Riyal with (*p* < 0.001). There were significant differences to suggest that the proportion of females’ nurses (94.8%) had intention to turnover more than male nurses (85.7%) and *p* value = 0.031.	-Education Access and Quality. -Economic Stability. -Social and Community Context.
[37]	Saudi Arabia 2023 (Makkah Region)	Quantitative cross-sectional study. Tool Used: A self-administered questionnaire	The study conducted on (n = 77 nurses) in the National Guard PHCs in the Makkah region, Saudi Arabia. Male: 54 (70%) Female: 23 (30%) Ages: 20 to over 50. Nationality: Saudi (n = 49), and non-Saudi (n = 18)	-Income level.	The mean satisfaction score for those receive < 10,000 SR (M = 3.04, SD = 1.07) is lower than those receive > 10,000 SR (M = 3.11, SD = 0.79). The estimated mean satisfaction score is 0.07. (*p* = 0.74) No significant difference.	-Economic Stability.
[38]	Saudi Arabia 30 May to 15 June 2020. (Hail Region)	Cross-sectional descriptive study design Tool Used: An online self-reported questionnaire	A total of 639 non-Saudi nurses participated from eight tertiary hospitals under the Ministry of Health in this study. Male: 17 (2.7%) Female: 622 (97.3%) Ages: 20 to over 50.	-Wages structure. -Level of education. -Years of experience. -Gender.	In terms of wages, the mean satisfaction score of nurses who had diploma holders (M = 3.4, SD = 0.9) is lower than that those who bachelors and other higher degrees (M = 3.9, 0.9). The estimated mean difference is 0.5. (*p* < 0.001). Significant difference. The mean score of intention to turnover of nurses who had diploma holders (M = 3.1, SD = 1.0) is lower than that of those who had bachelors and other higher degrees (M = 4.0, SD = 0.9). The estimated mean difference is 0.9. (<0.001). Significant difference. The results revealed that the foreign nurses who have experience for 0–4 years in tertiary hospitals were less satisfied (0.002) than other nurses who have long experience range from 5–10 years. The mean score of intention to turnover for male nurses (M = 3.2, SD = 1.0) is higher than for female nurses (M = 2.6, SD = 1.0). The estimated mean is 0.6. (*p* = 0.042). Significant difference.	-Economic Stability. -Education Access and Quality. -Social and Community Context.
[39]	Saudi Arabia 2018 (Tabuk Region)	Study applied a combination of descriptive, correlational, and cross-sectional analysis. Tool Used: A job satisfaction survey	A total of 190 critical care nurses at King Khalid Hospital in Saudi Arabia were recruited in this study. Male: 25 (13%) Female 165 (87%) Ages: 20 to over 50. Nationality: Not determined.	-Level of education. -Gender.	-The difference in the level of satisfaction was not statistically significant by the level of education (diploma, bachelor, and master) (*p* = 0.83). -There is no significant difference between males and females on the level of job satisfaction (*p* = 0.3).	-Education Access and Quality. -Social and Community Context.
[40]	Saudi Arabia 2022 (Hail Region)	A cross-sectional study Tool Used: Anonymous self-administered questionnaire	The study conducted in two large public hospitals in Hail City on 196 nurses. Male: 2 (1%) Female: 194 (99%) Ages: 18 to over 55. Nationality: Non-Saudi nurses.	-Pay.	Based on multiple linear regression tests, factor found to be one of the most significant causes of expatriate nursing turnover is pay, (*p* = 0.022).	-Economic Stability.
[20]	Saudi Arabia Between April and May 2018 (Riyadh)	Cross-sectional study. Tool Used: A self-administered structured questionnaire	Participants recruited from two public hospitals (n = 318) nurses. Male: 34 (10.7%). Female: 284 (89.3%). Ages: Mean 29.05 years old (SD = 3.30). Nationality: Saudi (n = 135), non-Saudi (n = 183).	-Marital status. -Nationality.	The mean score of intention to leave for single nurses (M= 3.27, SD = 0.68) is higher than for married nurses (M= 2.71, SD = 0.812). The estimated mean is 0.56. (*p* < 0.001). Significant difference. The mean score of intention to leave for non-Saudi nurses (3.34) is higher than for Saudi nurses (2.56). The estimated mean is 0.78.	-Neighborhood and Built Environment. -Social and Community Context.
[41]	Saudi Arabia Between April and May 2019 (Dammam)	Quantitative cross-sectional descriptive study. Tool Used: The Minnesota Satisfaction Questionnaire	A number of (n = 382) nurses working in a public hospital in Dammam city in Saudi Arabia were recruited in this study. Male: 45 (13.4%). Female: 292 (86.6%). Ages: 20 to over 40. Nationality: Saudi (n = 286), and non-Saudi (n = 51).	-Nationality. -Salary.	There was a significant difference in job satisfaction between Saudi and non-Saudi nurses. The mean satisfaction score of non-Saudi nurses (3.47) is 0.21 higher that than in Saudi nurses (3.26). There is no significant difference between job satisfaction and monthly salary (*p* = 0.038).	-Social and Community Context. -Economic Stability.
[42]	Saudi Arabia 2014 (Riyadh)	Cross sectional study. Tool Used: A self-administered questionnaire	A total of (n = 723) participated from a tertiary medical care center in Riyadh City. Male: 76 (10%). Female: 647 (89.5%). Ages: 30 to over 50. Nationality: Saudi (n = 46), non-Saudi (n = 677).	-Gender. -Nationality. -Level of education. -Salary.	The mean satisfaction score in male (103.43 ± 11.3) is lower than that in female (105.39 ± 8.2). The estimated mean difference is 1.96. (*p* = 0.15). No significant difference. The mean satisfaction score in Saudi nurses (102.9 ± 10.4) is lower than that in non-Saudi nurses (105.3 ± 8.5). The estimated mean difference is 2.4. The mean satisfaction score of those who have PhD (85.6 + 9.4) is lower than those who have Diploma (106.1 ± 8.5) and Bachelor’s (105.02 ± 8.4) (*p* < 0.001). Significant difference. The mean satisfaction score of nurses who were unsatisfied with payment (99.3 ± 9.3) is lower than that those who were very satisfied (109.5 ± 6.9). The estimated mean difference is 10.2. (<0.001). Significant difference.	-Social and Community Context. -Education Access and Quality.
[43]	Saudi Arabia 2022 (Riyadh)	Cross-sectional research design. Tool Used: Minnesota Satisfaction Questionnaire and Practice Environment Scale of the Nursing Work Index	A total of (n = 500) registered nurses working in five public hospitals in Riyadh. Male: 335 (67%) Female: 129 (25.8%) Do not want to disclose: 36 (7.2%) Nationality: Not determined.	-Gender. -Education.	The median job satisfaction score in those who did not want to disclose their gender (MD = 390.54) is higher compared to those who disclosed their gender as males (MD = 242.71) and females (MD = 231.66). (*p* < 0.001). Significant difference. The median job satisfaction score in those who had diploma holders (MD = 279.91) is higher compared to those who had bachelor (MD = 246.92) and postgraduate (MD = 148.29). (*p* < 0.001). Significant difference.	-Social and Community Context. Education Access and Quality.

### 2.5. Data Synthesis

To summarize and evaluate the body of evidence included in the review, a narrative synthesis was carried out using a textual and tabular approach. The results are reported in the next section. The discussion section reports the integration and interpretation of the results.

### 2.6. Quality Appraisal

The Joanna Briggs Institute (JBI) checklist tool was used to appraise the quality of the included studies [20,36,37,38,39,40,41,42,43] (Table 2). The quality reviews of all the papers were, however, independently evaluated by two evaluators (J.V. and A.H.). Any disagreements were settled by consensus and subsequently discussed with a third evaluator. The JBI tool indicates that studies are required to meet all the questions in the checklist. All nine studies, which were cross-sectional studies, failed to acknowledge all the confounding factors [20,36,37,38,39,40,41,42,43]. One study did not clearly provide criteria for the inclusion of the sample [42]. One study did not clearly mention the study setting [38] (see Table 3).

## 3. Results

### 3.1. Characteristics of the Included Studies

The included studies were published between January 2014 and March 2023, all from Saudi Arabia, across different cities (Riyadh, Dammam, Makkah, Hail, and Tabuk). All studies were cross-sectional [20,36,37,38,39,40,41,42,43]. Various settings were represented: tertiary hospitals (n = 12), public hospitals (n = 11), and primary healthcare centers (n = 1). The sample size included a total of 3389 participants, including a range of 77–723 nurses. The following were used as determinants of nurses’ job satisfaction across the nine studies: salary, total work hours, level of education, marital status, and years of experience. The measured outcomes included the barriers to job satisfaction (levels of job satisfaction, intention to leave, and turnover) (see Table 3).

### 3.2. Participants and SDOH Domains Assessed

The appraisal of the five domains of SDOH revealed that the social and community context was the main and most comprehensive domain across the selected studies. In order of priority, economic stability, education access and quality were the second most assessed domains across the five studies. The neighborhood and built environment domain was only assessed in one of the nine studies and was the lowest priority among the assessed domains.

### 3.3. Methods Used to Assess

Studies used several tools for assessing the SDOH of nurses’ job satisfaction, including a self-administered questionnaire, an online self-reported questionnaire, a survey questionnaire, an anonymous self-administered questionnaire, the Minnesota Satisfaction Questionnaire, and the practice environment scale of the nursing work index.

### 3.4. Pay and Nurses’ Job Satisfaction

Salary is considered the most important motivator influencing job satisfaction for many employees. Five of the nine studies evaluated the impact of income level on nurses’ job satisfaction. Of these, three studies [36,38,40] examined the exposure measure on (n = 9 Saudi nurses and n = 1199 non-Saudi nurses) who worked in multisite tertiary and public hospitals in Saudi Arabia and received low levels of income ranging from 1000 to 3000 Saudi Riyals (SR) and reported a significant intention to leave their job. Two studies did not find any significant difference among those who received less than 10,000 SR compared to those who received more than a salary of 10,000 SR [37,41]. One of the studies linked the difference between the level of nurses’ job satisfaction and monthly income to their qualifications and reported that those who had a diploma were less satisfied with their salary than those who had Bachelor’s and other higher degrees [38].

### 3.5. Job Satisfaction and Gender Differences

Work satisfaction is an emotional feeling that changes as a result of several factors that are derived from individual characteristics, as well as the impact of the sociocultural environment [44]. Five of the nine studies addressed the difference between males and females in terms of their job satisfaction and intention to leave their work. Of these, two studies did not report any significant difference between males and females in the level of their job satisfaction [39,42]. Two studies evaluated the intention to turnover between male and female nurses. In one of the studies, there was a significant difference between female nurses and their intention to turnover compared to male nurses (*p* = 0.031) [36]. The other study found a significant difference between male nurses and their intention to turnover compared to female nurses (*p* = 0.042) [38]. Only one study reported a significant difference between those who did not want to disclose their gender and job satisfaction compared to those who disclosed their gender as male and female (*p* < 0.001) [43].

### 3.6. The Impact of Professional Experience on Nurses’ Level of Job Satisfaction

Two of the nine studies addressed the correlation between years of experience and nurses’ levels of job satisfaction. Studies conducted in tertiary hospitals (n = 994 non-Saudi nurses and n = 9 Saudi nurses) reported that nurses who had less than five years of experience had significantly lower job satisfaction compared to those who had 5–10 years of experience [36,38], in which *p* value was 0.002 and 0.025, respectively.

### 3.7. The Association between Nationality and Nurses Job Satisfaction

Nationality characteristics were addressed in three studies. Two of nine studies found a higher mean satisfaction score for non-Saudi nurses compared to Saudi nurses [41,42] with mean scores of 3.47 and 3.26, compared to 105.3 and 102.9, respectively. By contrast, one study found a higher mean score of intention to leave among non-Saudi nurses compared to Saudi nurses [20], with mean scores of 3.34 and 2.56, respectively.

### 3.8. Job Satisfaction of Nurses by Education Level

There was a difference in qualifications and educational levels among the nursing workforce in Saudi Arabia. However, only four of the nine studies addressed the relationship between job satisfaction and education level, three of which found a significant difference between job satisfaction and nurses with lower levels of education [38,42,43] in which *p* value was <0.001, <0.001, and <0.001, respectively. One study reported no significance [39].

### 3.9. Marital Status

Marital status characteristics were found in one of the nine articles that examined this variable. This study conducted by [20] found a significant difference between single nurses and their intention to leave, with *p* < 0.001.

## 4. Discussion

This systematic review provides a detailed summary of the up-to-date studies on the factors affecting nurses’ job satisfaction by utilizing the SDOH framework through nine cross-sectional studies published between 2014 and 2023 that explored factors affecting nurses’ job satisfaction in Saudi Arabian hospitals. The results of this review showed significant differences between pay, years of experience, nationality, marital status, and nurses’ job satisfaction. However, the results did not provide evidence regarding gender differences and educational attainment as predictors of nurses’ job satisfaction.

### 4.1. Pay and Nurses’ Job Satisfaction

There are similarities and differences between the current and other review findings that address salary determinants among nurses. According to the results of this review, there is a significant difference between low income ranging from 1000 to 3000 Saudi Riyals and nurses’ job satisfaction [36,38,40], which is consistent with the study conducted by [45], who reported a significant difference. The current review also included studies that contradict the previous findings [37,41], which did find a significant difference between salary and nurses’ job satisfaction. The disparity in salary structures distinguishes the two groups. Most of those dissatisfied with their salaries in our reviewed studies were non-Saudi nurses working in large and specialized hospitals. One of the reasons that might make them feel unsatisfied with their salaries is the inequality between them. There is no precise scale for salaries for those who come from abroad to work in the Saudi Arabia Ministry of Health hospitals, and their salaries are sometimes based more on nationality than on education level and experience. However, in a study conducted by Alshmemri [46], it was claimed that in Saudi hospitals, Malaysian nurses who had lower qualifications earn higher salaries than nurses from India and the Philippines who had higher qualifications.

Another factor is that nurses might need to work long hours (12 h) to cover duties related to the staff shortage without additional compensation, which negatively affects their physical and psychological status. According to Aljohani [47], such stress leads to a hostile work environment that places nurses at risk for chronic tiredness, poor physical performance, and inadequate interaction. These findings highlight the importance of the Saudi healthcare system addressing the economic stability determinants of health in improving Saudi and non-Saudi nurses’ job satisfaction. This finding is consistent with the SDOH framework, which addresses the importance of economic security, which is considered an essential factor in the health and well-being of families.

### 4.2. Job Satisfaction and Gender Differences

In terms of gender differences, our review found three studies with contradictory results. One study found that female nurses had a higher intention to leave than male nurses [36], unlike the second study, which reported that male nurses had a higher intention to leave than female nurses [38]. The third study found that those who did not want to disclose their gender had a higher level of perceived job satisfaction [43]. A previous study also found no significant difference in mean job satisfaction scores between male and female nurses [48]. By contrast, many studies have reported a significant difference between nurses’ gender and their levels of job satisfaction [49,50,51]. However, the results of these studies’ results are varied. For example, Al-Ahmadi’s study [49] found that female nurses had significantly lower job satisfaction than male nurses, including Saudi and expatriate nurses. Rajapaksa and Rothstein [50] conducted a study in the United States to examine the factors affecting male and female nurses’ decisions to leave work. They found a higher rate of leaving among male nurses than among female nurses.

Some factors might lead to this discrepancy in the results. First, the perceptions of males and females regarding job satisfaction may differ. Second, comparing male and female perceptions is meaningfully challenging when one group is much smaller than the other. For example, our review study examined the variable gender among 387 male nurses and 1080 female nurses. Based on these contradictory results and views, we cannot provide conclusive evidence about gender as a predictor of job dissatisfaction. Thus, further studies are needed to address this knowledge gap.

### 4.3. The Impact of Professional Experience on Nurses’ Level of Job Satisfaction

Regarding the years of experience, our review showed that those nurses who had experience ranging from 5 to 10 years of experience had significantly higher job satisfaction compared to those who had experience ranging from 1 to 4 years [24], which is inconsistent with the findings of Ma et al. [52], who indicated nurses who had less than two years of experience had a higher level of job satisfaction compared to those who had experience of more than two years. In addition, a study conducted by Kacel et al. [53] in the United States, which investigated the job satisfaction of nurse practitioners, found that nurses who had recently graduated and had less practical experience were more satisfied than other nurse groups.

By contrast, our findings are compatible with Al-Aameri [54] and Almalki et al. [55], who reported that nurses with fewer years of experience were less satisfied than those with more experience. It is worth noting that the two studies addressed the variable of the years of experience among 994 non-Saudi nurses and only 9 Saudi nurses. Therefore, expatriate nurses who had extensive experience and felt satisfied had good knowledge of Saudi culture. Nurses who had fewer years of experience lacked an understanding of Saudi patients’ culture. By understanding the patient’s cultural background, nurses can help provide support and optimal healthcare, as it also helps to avoid misunderstandings between nurses, patients, and their families. Nevertheless, the SDOH framework explained the results well [29]. The findings showed how important it is for the Ministry of Health to promote and encourage foreign nurses’ education while exposing them to Saudi Arabia’s cultural and religious practices in healthcare delivery.

### 4.4. Nationality and Nurses Job Satisfaction

The current review findings of two studies assessed the relationship between job satisfaction and nationality and revealed that non-Saudi nurses had higher mean satisfaction scores than Saudi nurses. Many expatriate nurses leave their countries looking for a place to practice. These nurses look for work in developed countries, as they are well-trained, and most have experience and work in their own countries. However, some may leave their countries and families to work overseas due to economic crises. The prior studies of Al-Aameri [54], Al-Ahmadi [56], and Adams and Bond [57] are incompatible with the present findings, as they indicated that nationality did not affect job satisfaction.

It is worth noting that our studies were conducted in two major cities in Saudi Arabia (Riyadh and Dammam). These two cities also have many Saudi nurses who left their original hometown and their families, and many of them pay rent for housing and do not have a means of transportation compared to non-Saudi nurses, who have free housing from their sponsor, which is the Ministry of Health, as well as secure means of transportation. However, these factors could be investigated further with the SDOH framework [29].

### 4.5. Job Satisfaction of Nurses by Education Level

The current review found that those nurses who had (diplomas) were more satisfied than those who had higher levels of education (BSN, MSN, and Ph.D.), which is incompatible with Rambur et al. [58] and Tzeng [59] and Yin and Yang [60], who reported higher job satisfaction for those nurses who held higher educational levels than for those who had lower educational levels.

By contrast, our findings are consistent with Al-Ahmadi [49] and Lu et al. [26], who found that nurses with Bachelor’s degrees demonstrated a high level of turnover intention and dissatisfaction, more so than those with lower levels of education. Moreover, Dunn et al. [61], Fang, [62], Hu and Liu [63], and Larrabee et al. [64] did not find any significant difference between job satisfaction and educational level.

Based on these contradictory results, there is no consensus regarding educational attainment and its relationship with job satisfaction or intent to leave. The inconsistency in the results may be attributed to the fact that researchers have similarities in the method of data collection and analyzing data using different statistics, which hinders elucidating the reasons for the differences in the results. Therefore, this is a gap in the literature that needs further investigation.

### 4.6. Marital Status

We found a significantly higher mean score of intention to leave among single nurses than for married nurses. Our findings agree with a study by Ma et al. [65] in acute care hospitals in Taiwan, which reported that 71% of single nurses had the intention of leaving their jobs. Additionally, one study found that young single nurses were more likely to contemplate quitting their jobs than older nurses [58]. Moreover, our findings are consistent with the studies conducted by Masum et al. [66], Top and Gider [67], and Torkelson and Seed [68], who found that single nurses had a higher intention to quit their current workplace than for married nurses.

The independent variable, marital status, was associated with social support beliefs because married individuals received moral and social support from their families or co-workers more than unmarried individuals. Undoubtedly, one of the essential factors in nurses’ professional status is marital satisfaction, which helps improve and decrease the work burden through continuing support and can, thus, enhance their quality of life, which reflects positively on their health status. This claim is in line with Amponsah’s [69] findings, which indicate that a marriage can help prevent job stress and increase marital happiness. Therefore, policymakers in the Ministry of Health should develop strategies or solutions for improving social support for single nurses to ensure that nurses can be more satisfied. The results are well explained within the SDOH framework [29], as they highlight the necessary social cohesion among nurses, their families, and co-workers and how that might impact their job satisfaction.

### 4.7. Strengths and Weaknesses

The systematic review identified factors that led to job dissatisfaction and revealed nurses’ perceptions. The review is important in that it has been carried out when limited studies highlight social determinants related to nurses’ job satisfaction in Saudi Arabian hospitals. Based on the JBI tool that was used, the included studies were of high quality, which allows for the generalization of the findings in Saudi Arabia. Furthermore, our review focused only on studies conducted in Saudi Arabia.

Points of weakness in the review include some studies that lack resemblance in the subjects’ characteristics, which may reduce the validity of these results. Furthermore, the small sample sizes of certain studies made it more difficult to find significant differences in our review.

### 4.8. Limitations

It is necessary to consider some limitations when interpreting the findings of this review. The review covered only papers published in English, which may have resulted in non-English studies being overlooked. Consequently, some relevant articles in other languages might have been missed. Given that this review was conducted in such a short amount of time and just three databases were searched, other relevant studies were likely missed. This review included a small number of articles (n = 9), which might limit the findings to be generalized. Moreover, all included studies were cross-sectional, which might limit the ability to estimate causation and decrease the generalizability of the results. Furthermore, most of studies relied on self-reported data, which indicate measurement bias; hence, this may affect the validity and reliability of the findings presented in this review. Although there is a significant body of research on nurse work satisfaction, this systematic review is the first of its kind in the Saudi Arabian setting.

## 5. Conclusions

This systematic quantitative review analyzed nine studies on the factors affecting nurses’ job satisfaction using the SDOH model. In the included studies that were scrutinized for the purpose of the current study, it was found that salary, years of experience, nationality, and marital status were factors affecting nurses’ job satisfaction. There are some areas where there is a lack of knowledge regarding the effect of gender and educational levels on job satisfaction among nurses. This knowledge gap needs to be addressed through further research. However, the current findings may provide nurses working in Saudi Arabian hospitals with suggestions or solutions for addressing SDOH in clinical contexts and beyond, supporting system and policy reforms that acknowledge SDOH. Moreover, these findings provide insights for decision-makers in designing interventions to influence the SDOH of nurses’ job satisfaction and, thus, promote prompt solutions.

## Figures and Tables

**Table 1:** Summary of literature search based on inclusion criteria.

Inclusion Criteria	Exclusion Criteria	Key Words	Databases Searched	Number of Studies
Studies that are of a quantitative type.	Systematic Review.	Saudi Nurses OR Staff Nurses OR Head Nurses OR Nurses Managers OR Undergraduate Nurses OR Postgraduate Nurses OR Saudi Nurse* AND Social Determinants of Health OR Cultural Issues OR Social Barriers OR Educational Barriers OR Environmental Factors OR Economical Factors OR Health Organization Challenges OR Determinants of Health OR SDOH* AND Job Satisfaction OR Work Satisfaction OR Workplace Satisfaction OR Satisfaction OR JS*	PubMed	113
Participants were Saudi and non-Saudi nurses.	Studies conducted outside Saudi Arabia		PsycINFO	60
Studies settings should be at Saudi healthcare setting.	Studies that focus on other health profession.		CINAHL	45
Studies written in English language.	Books.		Google Scholar	17
Studies published in the last 11 years.	Unrelated language.			
				Total: 235
	Studies published before 2012.			

**Table 3:** JBI checklist for cross-sectional studies.

Checklist Questions	[42]	[36]	[39]	[20]	[41]	[38]	[43]	[40]	[37]
Were the criteria for inclusion in the sample clearly defined?	Unclear	Yes	Yes	Yes	Yes	Yes	Yes	Yes	Yes
Were the study subjects and the setting described in detail?	Yes	Yes	Yes	Yes	Yes	Unclear (setting)	Yes	Yes	Yes
Was the exposure measured in a valid and reliable way?	Yes	Yes	Yes	Yes	Yes	Yes	Yes	Yes	Yes
Were objective, standard criteria used for measurement of the condition?	Yes	Yes	Yes	Yes	Yes	Yes	Yes	Yes	Yes
Were confounding factors identified?	Unclear	No	No	No	No	No	No	No	No
Were strategies to deal with confounding factors stated?	Unclear	No	No	No	No	No	No	No	No
Were the outcomes measured in a valid and reliable way?	Yes	Yes	Yes	Yes	Yes	Yes	Yes	Yes	Yes
Was appropriate statistical analysis used?	Yes	Yes	Yes	Yes	Yes	Yes	Yes	Yes	Yes

## Data Availability

Data are contained within the article.
